# Evaluation of the Stability of Polymeric Materials Exposed to Palm Biodiesel and Biodiesel–Organic Acid Blends

**DOI:** 10.3390/polym10050511

**Published:** 2018-05-09

**Authors:** Libia M. Baena, Ernesto C. Zuleta, Jorge A. Calderón

**Affiliations:** 1 Grupo de Calidad, Metrología y Producción, Instituto Tecnológico Metropolitano ITM, Medellín 050034, Colombia; 2 Grupo de Procesos Fisicoquímicos Aplicados-PFA, Universidad de Antioquia, Medellín 050034, Colombia; eczuletas@gmail.com; 3 Centro de Investigación, Innovación y Desarrollo de Materiales-CIDEMAT, Universidad de Antioquia, Medellín 050034, Colombia; andres.calderon@udea.edu.co

**Keywords:** biodiesel, polymeric materials, degradation, mechanic properties, fatty acids

## Abstract

The aim of the present work is to evaluate the impact of pure palm biodiesel fuel (B100) and biodiesel blends with 0.32% oleic, palmitic, acetic, myristic, and stearic acids on the properties of some polymeric materials used commonly in the manufacture of auto parts such as the polyamide 66 (PA66), polyoxymethylene (POM), and high-density polyethylene (HDPE). The effects of the B100 and B100–acid blends on polymeric materials were examined by comparing changes in the gain/loss of mass and by measuring the hardness, the impact strength, and the tensile strength of the materials at the end of the exposure. The characterization of the polymers was carried out before and after exposure by using differential scanning calorimetry (DSC), thermogravimetric analysis (TGA), and Fourier transform infrared spectroscopy (FTIR). After the immersion in B100–acids blends, the HDPE exhibited an increase in mass of 5%, which was very similar in all blends. The PA66 showed a small decrease in weight (2% approx.) in all mixtures. The POM presented an increase in the percentage of weight in the mixture of B100 with acetic acid of 0.3%. A decrease was observed in the crystallinity of the HDPE when exposed to blends of B100–acids. This behavior may be associated with a plasticizing effect in the HDPE exposed to the blends. The mechanical properties of POM and HDPE showed no significant changes after immersion in the fuels. On the other hand, PA66 exhibited a significant decrease in maximum stress value after immersion in B100, B100–oleic acid and B100–palmitic acid blends. The variation of the mechanical properties of the PA66 after exposure to B100 was potentiated by addition of organic acids. The assessed polymers did not undergo appreciable changes in the chemical structure of the samples after immersion in the fuels, so the variation in the mechanical properties could be explained by physical absorption of the fuel into the polymers.

## 1. Introduction

Biodiesel—a fuel produced from vegetable oils, animal fats, or used cooking oils—has been employed in many countries as a replacement for fossil fuels for use in diesel engines, mainly in transportation and cargo trucks. Because of the environmental advantages that this biofuel presents (e.g., renewability, reduction of CO_2_ emissions), it has been introduced as a greener alternative that can also incentivize industrial development through the implementation of an agri-energy economy around its production [1].

The principal vegetable oils used to produce biodiesel are jatropha, colza, corn, palm, cotton, sunflower, soybean, and coconut oil. Waste vegetable oil from restaurants, food industry, and residential uses has been also used to produce biodiesel [2]. Since the alkyl esters that form biodiesel (methyl esters) have properties similar to traditional diesel, it can be mixed with petroleum diesel in different proportions without compromising the quality of the fuel (usually 10 to 20%) [3]. As a result, it is not necessary to make appreciable modifications to the structure of diesel engines and storage tanks when biodiesel is present in the diesel fuel [4]. Nevertheless, there are some concerns related to the exposure of the parts and materials in the fuel transportation and ignition system in the engines that can be in contact with the biodiesel.

One factor that can potentially contribute to the deterioration of these materials is the presence of acidity, like free fatty acids (FFA), which are present in the triglycerides (oils are mainly triglycerides) and can be also found in the biodiesel [3,5,6,7,8]. One important source of FFA is the hydrolysis reaction that affects the methyl esters. The presence of water in biodiesel—from the purification process or absorbed during fuel storage and distribution—can promote the apparition of hydrolytic reactions. The presence of residues of basic catalysts (that can also catalyze the hydrolysis), such as mono- and diglycerides, glycerol, and alcohol from the transesterification reaction, can also drive the formation of FFA in the biodiesel and increase the water affinity of the biodiesel [5,9,10,11]. Another source of acidity in biodiesel is associated with the oxidation that it can suffer. As it has been established, one characteristic of biodiesel is the poor oxidative stability of the unsaturated fatty acids that comprise the methyl ester [12,13]. The presence of radical initiators, such as trace metals, peroxides, heat, and light, can conduce to the oxidation of the unsaturated methyl esters. The biodiesel oxidation is a radical-chain multistep reaction that breaks down the unsaturated methyl ester, leading to a rapid formation of hydroperoxides and a series of byproducts, including organic acids, that can compromise the material in contact with the biofuel [14,15,16,17]. It has been reported that FFA and organic acids are responsible for the degradation and deterioration of biodiesel and therefore make it an incompatible fuel with some materials used in the manufacture of auto parts [18]. Among the materials affected by biodiesel are polymers, which reflect their incompatibility with biodiesel by presenting swelling and deterioration of their physical, chemical, and mechanical properties. The swelling of the polymer is the increase of its volume, generated by the interaction with a solvent that is retained by the polymer when the latter has chemical affinity with the biodiesel, which depends on fuel composition and formulation polymer [19].

Besides the swelling, biodiesel can also cause structural changes in volume and mass, and changes in mechanical properties such as elongation, hardness, and tensile stress in polymers [20]. A recent study examined the performance of HDPE in soybean oil and sunflower oil biodiesel by static immersion test at 60 °C for 125 days observing a significant weight gain of about 5% [21]. Gary et al. [22] found that some physical properties of polymeric materials, such as nylon 6/6, nitrile rubber, and high density polypropylene, were affected after being immersed in biodiesel blends at 51.7 °C for 694 h. Teflon, Viton A401-C, and Viton GFLT had insignificant changes in physical properties after exposure to biodiesel. Haseeb et al. [20] investigated the impact of palm biodiesel on degradation and deterioration of nitrile rubber (NBR), polychloroprene, and fluoro-Viton A by static immersion test at 50 °C for 500 h. They observed that there were no significant changes in the volume, mass, tensile stress, elongation, and hardness for the three polymers tested. Another study compared the degradation of elastomeric materials—such as silicone rubber (SR), polytetrafluoroethylene (PTFE) and ethylenepropylenediene monomer (EPDM) exposed to different mixtures of palm biodiesel and diesel at 25 °C for 1000 h—by checking the changes in physical and mechanical properties. The PTFE showed a reduction in mass and volume, while EPDM presented a weakening in hardness and tensile stress in mixtures with higher concentrations of biodiesel [23]. Liguang et al. [24] observed changes in mass and diameter lower than 1% for polyethylene (PE) and polyoxymethylene (POM) after the immersion in biodiesel of palm, canola, soybean, and cotton at room temperature for 28 days. Therefore, it is of great importance to evaluate the performance against degradation and the ways to reduce the deterioration of the materials used in auto parts by palm biodiesel produced in the country. The use of palm biodiesel represents approximately 30% of all biodiesel produced in the world. For instance, the total use of palm oil in the EU grew by 1.6% to 7.3 million tonnes in 2015, and its usage continues to grow until now. However, the participation of palm biodiesel in the biodiesel consumption of a country depends largely on its geographical conditions to produce it or on its prospects of importing it. Colombia, being a tropical country, is a producer of, and is self-sufficient in, palm biodiesel; the biodiesel consumed in Colombia is 100% palm biodiesel. So, the large amount of palm biodiesel consumed in the world, and particularly in Colombia, justifies this study. This work aims to analyze the impact of palm biodiesel and its blends with fatty acids and acetic acids on the properties of three polymeric materials that are commonly found in the fuel system of cars, that is, polyoxymethylene (POM), high density polyethylene (HDPE) and polyamide 66 (PA 66).

## 2. Materials and Methods 

### 2.1. Materials

In this study, samples of three polymeric materials that are commonly used in auto parts and that are in direct contact with the fuel in real use were selected. Square samples of polyamide 66 (PA66), polyoxymethylene (POM), and high-density polyethylene (HDPE) supplied by a car manufacturer (Medellin, Colombia) were taken with an exposed area of approximately 2 cm^2^ and 3 mm of thickness. Those materials are commonly used in different auto parts such as the canister, the housing of the fuel pump, and the fuel tank.

### 2.2. Preparation of the Fuel and Its Blends with Acids

The biodiesel used in this study was made by basic transesterification of palm oil (Oleoflores, Agustin Codazzi, Colombia) and methanol (Fisher Scientific, Hampton, NH, US), using KOH as catalyst. The process included a transesterification reaction, separation of the glycerin, and washing with hot water. Finally, the biodiesel was dried under vacuum. The biodiesel was characterized using gas chromatography (GC) to evaluate the methyl ester composition, which is presented in Table 1. The methyl ester content analysis was done following the EN 14103 standard, using a gas chromatograph Agilent 7890A (Santa Clara, CA, USA) with a capillary column Agilent J & W HP-Innowax, a flame ionization detector, and tetradecanoic acid as internal standard. The characterization of the biodiesel showed that the methyl ester content of the neat biodiesel fuel (B100) was above 97% (mass/mass). It had a balanced composition of saturated and monounsaturated methyl esters of 43.3% (C16:0) and 41.8% (C18:1), respectively. The physicochemical properties of tested palm biodiesel are shown in Table 2.

**Figure 1:**
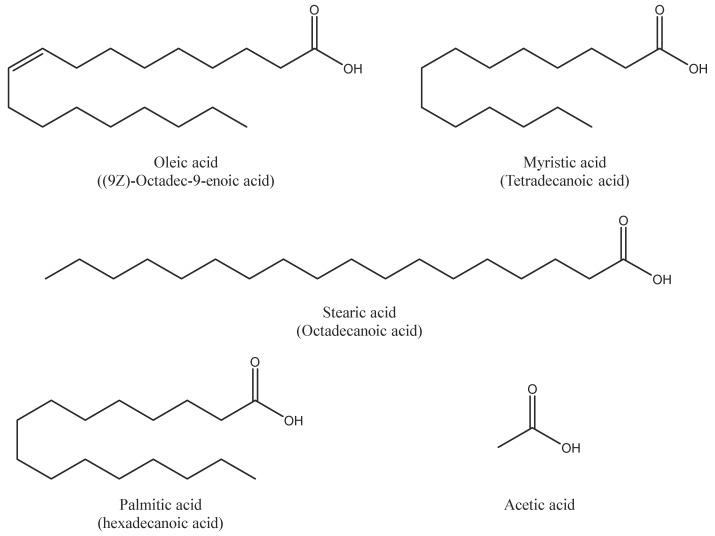
Chemical structures of the acids added to the biodiesel.

In order to evaluate the effect of organic acids on polymeric materials in contact with biodiesel, we proceeded to make four binary blends of B100 and fatty acids, and an additional blend of B100 and acetic acid. The fatty acids added to the biodiesel were: oleic, myristic, palmitic, and stearic acid; all of them from Merck KGaA (Darmstadt, Germany). The B100–acetic acid blend was also tested because acetic acid is one of the more common acid species formed in biodiesel oxidation reactions and it could be aggressive to the materials. Figure 1 shows the chemical structure of the acid species used in the preparation of blends, and Table 3 presents its physical and chemical characteristics. The U.S. standard ASTM D6751 was followed as a reference to select the amount of fatty acid and acetic acid to add to the fuel blends. The standard states that the maximum acid concentration in biodiesel should be 0.8 mg KOH/g (TAN), the value equivalent to 0.32% of fatty acid (evaluated as palmitic acid for palm oil biodiesel). The total acid number (TAN) is the number expressed in milligrams (mg) of potassium hydroxide required to neutralize one gram of acid present in biodiesel, thus determining the level of free fatty acids generated in the production of biodiesel or from oxidation reactions [9].

Consequently, the amount of acid species was added to biodiesel in this proportion. Therefore, the biodiesel–acid blends with 0.32% of acid species became the worst-case scenario to evaluate the aggressiveness of the fuel in contact with polymeric materials.

### 2.3. Analytic Characterization

In order to assess the impact of the neat biodiesel and biodiesel–acid blends on the polymeric materials, static immersion tests were conducted following the procedures described in the SAE J1748 standard [25]. Six samples of each material were totally immersed in B100 and B100–acid blends in different glass vessels at a controlled temperature of 55 °C. The weight of the samples was registered weekly until its stabilization, normally reached after 14 weeks for polymers exposed to B100 and 19 weeks for polymers exposed to B100–acid blends. After this time, the samples were withdrawn for characterization. Weight measurements were performed using a laboratory balance of four significance digits. The volume of the polymers during the immersion test was also monitored; however, there were no appreciable changes in volume. For this reason, it is not reported. In order to corroborate physical and chemical interactions between the fuels and the polymer materials, thermal analyses were performed by differential scanning calorimetry (DSC) using a thermal analyzer DSC Q200 (TA Instruments, New Castle, DE, US) with a heating rate of 20 °C min^−1^ in a temperature range of 20–400 °C in nitrogen atmosphere. Thermal decomposition studies were conducted in a temperature range of 20–800 °C with a TGA Q500 V20.8 Build 34 0500-1190 Serial instrument (TA Instruments, New Castle, DE, USA) at 20 °C min^−1^ with a nitrogen flow of 60 mL min^−1^. The thermogravimetric analysis was performed in order to achieve the decomposition of the material and obtain the maximum degradation temperature. Also, the absorption of some compounds into the material can be monitored by the TGA analysis. The temperature of degradation of the polymer can be change by interaction with the environment—in this case, with the fuel. The interaction of the polymer materials with the fuel could introduce changes in the chemical or physical structure of the polymer, which leads to changes in the thermal properties of the materials. In order to determine the chemical structural changes in the polymers, Fourier transform infrared (FTIR) spectra analysis was performed in the wavenumber range of 4000–400 cm^−1^, in a Shimadzu IR Prestige Model 21 Infrared Spectrophotometer (Kyoto, Japan).

Before and after the immersion of the samples, hardness, impact resistance, and stress–strain tests were performed on polymeric materials in accordance with the standard test method for plastic properties. The hardness tests were done in accordance with the procedures established in the ASTM D2240-05 standard [26]. The impact resistant was evaluated by the Charpy method (ISO179-1997 [27]), while the tensile strength, fracture stress, and maximum elongation values were obtained from the stress–strain curves (ASTM D638-03 [28]). It is important to mention that fracture stress, also known as fracture strength, is the stress at which a specimen fails via fracture. This is usually determined for a given specimen by a tensile test, which charts the stress–strain curve. The final recorded point, when material fails by fracture, is the fracture stress. These tests were conducted with the aim of detecting changes in the mechanical properties of the materials due to the physical or chemical interactions between the fuel and the polymeric materials. The impact resistance and stress–strain tests were performed only in B100 and B100-blend modified with the addition of 0.32% palmitic and 0.32% oleic acids (modified). The mixture of B100 with oleic acid and palmitic acid was employed in these tests because those two fatty acids are found in greater proportion in palm biodiesel and because oleic acid is an easily oxidizable compound. It could negatively affect the mechanical properties of the materials under study.

## 3. Results and Discussions

### 3.1. Mass Variations of the Polymeric Materials after Immersion in Biodiesel and Blends

**Figure 2:**
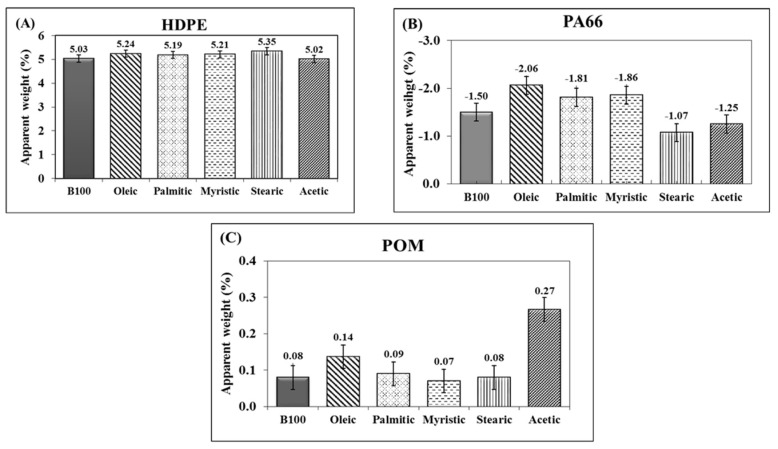
Variation of apparent weight of polymeric materials after immersion in pure palm biodiesel (B100) and B100–acid blends. (**A**) HDPE, (**B**) PA66 and (**C)** POM.

Figure 2 shows the variation of the apparent weight of the polymers exposed to pure biodiesel (B100) and biodiesel–acid blends. As it can be seen in Figure 2A, the HDPE presented a similar mass increase in all the tested media. During the 10 first days of immersion, HDPE samples reached a weight increment of approx. 5%, keeping this weight until the end of the test. Apparently, it is at this time that the polymer reaches its saturation—that is, the polymer cannot absorb more fuel, so further increases are not expected. According to the results given by the gain/loss mass test, it could be concluded that the addition of acid species in palm biodiesel did not induce additional absorption of fuel, other than that caused by pure B100, into the HDPE. Figure 2B shows the variation of the apparent weight of PA66 samples exposed to the fuels. After exposure to B100, B100–acetic, and B100–stearic acids blends, the PA66 samples presented a total weight loss of about 1.5%, whereas in B100–myristic, B100–oleic, and B100–palmitic acid blends, the weight loss was near 2%. It was observed that the weight loss occurred mostly during the first 2 weeks of immersion. After that time, the samples maintained a constant weight until the end of the test. These small reductions in weight are related to the leaching of plasticizers and additives added to the polymer to prevent hardening [29,30]. It can be seen in Figure 2C that the POM did not show any significant differences in mass after immersion in the fuel blends, exhibiting small mass increases close to 0.1 wt % in B100 and B100–acid blends, during the first 8 weeks of exposure. After that, mass differences were constant until the end of the test. This small increase in weight may be associated with the absorption of polar compounds present in the biodiesel due to the affinity that this material possesses with the ester groups [8,20] and polar compounds of the acid species, like acetic acid.

### 3.2. Differential Scanning Calorimetry (DSC) Study

**Figure 3:**
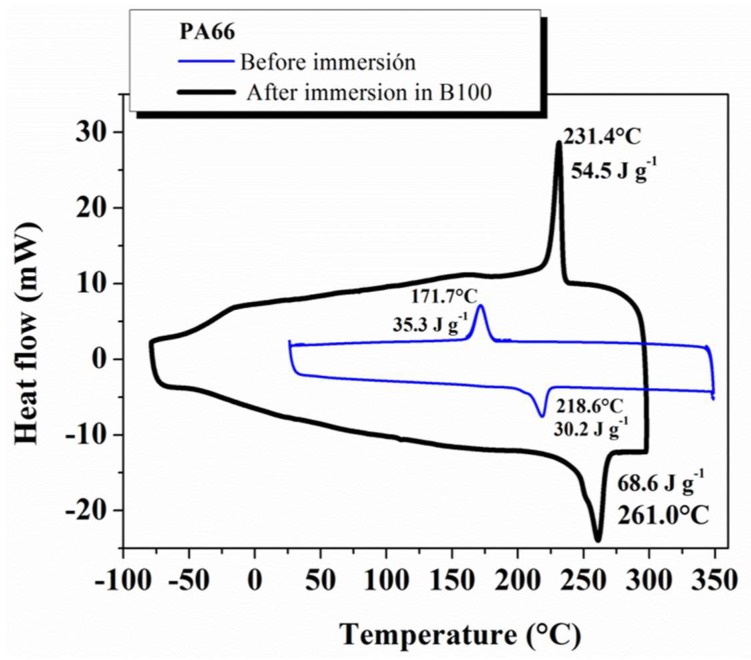
Differential scanning calorimetry (DSC) thermograms of the polyamide 66 (PA66) polymer before and after immersion in B100.

Figure 3 shows the DSC measurements of the PA66 sample before and after immersion in B100 (14 weeks). Significant changes in the melting and the crystallization temperatures of the PA66 were observed after immersion. The DSC curves of the PA66 samples show the fusion process for both before and after immersion as well-defined endothermic peaks (melting temperature) at 218.64 °C and at 260.69 °C, respectively. Similarly, during the cooling process, the crystallization peaks of the PA66 samples before and after immersion in B100 were observed at 171.73 and 231.36 °C, respectively. These results suggest that immersion in B100 causes a significant increment of the fusion and crystallization temperatures of the polymer. An analysis of the degree of crystallinity of the PA66 samples before and after immersion in B100 was performed using Equation (1) [31].

(1) $$Xc=\frac{\Delta Hx}{\Delta Hm{}^{\circ}}\times 100$$

where Δ*Hx* is the enthalpy of the process (fusion or solidification) calculated from DSC test and Δ*Hm*° is the theoretical value of the enthalpy of the 100% crystalline PA66, taken as 196 J g^−1^ [32]. The crystallinity of the PA66 samples changed from 15.4% to 35% after immersion in B100. This result indicates that the increment in the crystallinity of the material happened due to interaction with the fuel. It was corroborated that the crystallization of the polymer starts at higher temperatures and involved larger enthalpies when the material is immersed in B100 (*T*c = 231.36 °C and Δ*Hx* = 54.48 J·g^−1^). The PA66 behavior can be explained by the fact that it interacts with polar compounds present in the biodiesel that are able to form intermolecular bonds such as hydrogen bonds due to the presence of the amide group (NHCO), which is a highly polar group. When PA66 forms hydrogen bonds, it acquires a more rigid structure. Therefore, the flexibility of the chains is reduced, leading to an increase in its melting temperature [33]. The crystallinity of a polymer is the result of ordered molecular aggregation, with molecules held together by secondary valence bonds. Secondary bond forces are responsible for intermolecular bonding in polymers [33]. The tendency of a polymer to crystallize depends on the extent of intermolecular binding forces and their structural features. Therefore, crystallinity may be viewed as a form of physical cross-linking which is thermoreversible. If a polymer possesses significant intermolecular forces, it becomes as a crystallizable material. Consequently, it will have high melting points and becomes more rigid [31]. The melting temperature generally increases in line with crystallinity. Therefore, with the formation of hydrogen bonds in the PA66, the degree of crystallinity of these materials and their melting temperatures increase [33]. An additional effect on the melting temperature increasing could be related with the plasticizer leaching after immersion in the fuels.

**Figure 4:**
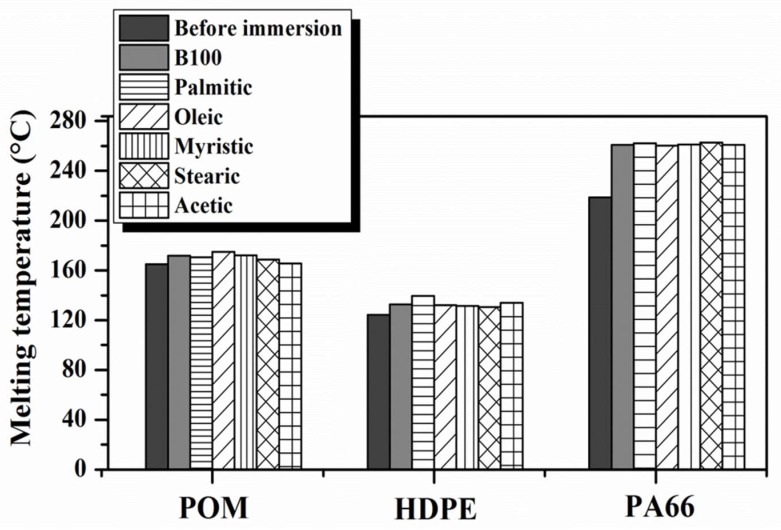
Melting temperature variation during DSC analysis for the polymers after immersion in B100 and B100–acid blends.

**Figure 5:**
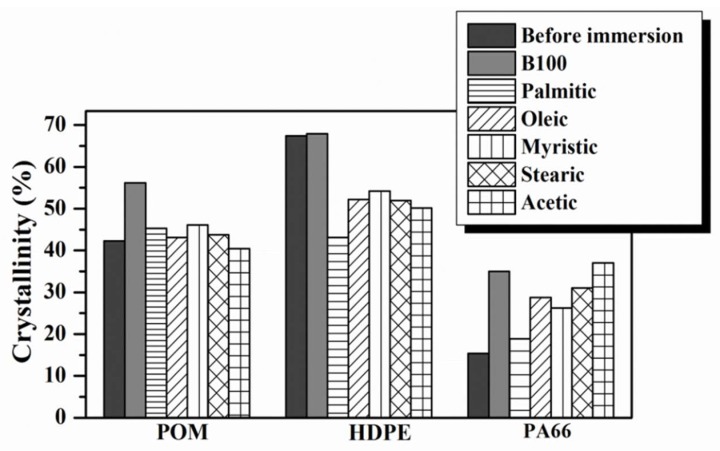
Variation in the crystallinity of the polymer materials after immersion in B100 and B100–acids blends.

The rise in cohesive energy density (Δ*H*) in the PA66, from 30.18 J·g^−1^ before immersion to 68.60 J·g^−1^ after immersion in B100, is also associated with the rise in the density of sites for intermolecular bonding (space decreases between polar groups), which leads to an increase in the melting points. Likewise, DSC thermograms were also obtained and analyzed for HDPE and POM polymers before and after immersion in B100 and B100–acid blends. The compiled results of the melting temperatures and degree of crystallinity of all tested polymers are shown in Figure 4 and Figure 5, respectively.

A minor increase of the melting temperatures of the POM and HDPE samples after immersion in B100 and B100–acid blends was observed, but much less than those observed for PA66 polymer. Polymers with rigid chains would be expected to have higher melting points than those with flexible molecules due to polymers with stiff backbones having lower conformational entropy changes than those with flexible backbones [33]. The flexibility of the HDPE chain was slightly affected by the presence of new carbonyl groups formed after interaction with the biodiesel, which will be explained later. The insertion of these new carbonyl groups restricts rotation of the chain and consequently reduces the conformational changes of the chain, resulting in a slight increase in melting temperature of the polymer (see Figure 4). The addition of the acid to B100 does not induce significant changes in the melting temperatures of the POM and HDPE polymers.

On the other hand, an important effect of the acid addition was observed in the crystallinity degree of HDPE. The degree of crystallinity was always lower for the HDPE samples exposed to B100–acid blends than those only exposed to B100. This behavior may be explained by the fact that, as was observed in Figure 1, the HDPE sample exhibited the largest absorption of fuel after exposure in comparison with the other tested polymers. The absorbed biodiesel blend, which is trapped between the HDPE organized chains, acts as a plasticizer and makes them slide, causing the reduction of the crystallinity of the material. In the case of POM, being a polymer partially having a polar group –O–, strong secondary bonds (such as hydrogen bonds) form when interacting with biodiesel, which causes a gentle increase in their melting temperature and crystallinity (see Figure 4 and Figure 5) [33].

### 3.3. Thermogravimetric Analysis (TGA)

**Figure 6:**
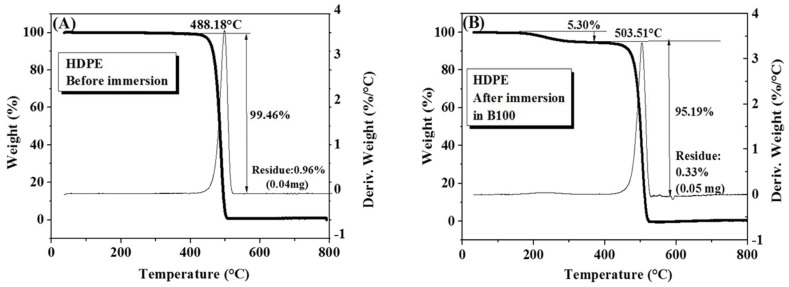
Thermogravimetric analysis (TGA) curves of high-density polyethylene (HDPE) samples. (**A**) Before immersion; (**B**) after immersion in B100.

Figure 6 shows the thermogravimetric curves and their derivatives for HDPE samples before and after immersion in B100 for 14 weeks. Figure 6A corresponds to the thermogram of HDPE sample before immersion. A significant mass loss of 99.46% due to thermal degradation at 488.18 °C can be seen [34]. At the end of the test, a residue of 0.96% was obtained. Figure 6B shows the TGA curve of the HDPE sample after immersion in B100. A mass loss of 5.30% starting from 200 °C is initially observed, which is associated with biodiesel absorption due to its affinity with this material. The samples immersed in B100 showed a slightly larger thermal stability than samples before immersion, exhibiting a mean degradation temperature of 503.51 °C, with a mass loss of 95.19% and a residue of 0.33%. Comparing the two thermograms of HDPE samples, before and after immersion, it is possible to observe three stages of decomposition. At around 200 °C, the first stage, some components evaporate. In this case, it corresponds to removal of the fuel absorbed. At temperatures from 400 to 500 °C (second stage), major chains quickly decompose and form thermally stable char. Above 500 °C, the last stage, the char slowly decomposes. According to the results of these thermograms, it can be said that there are no significant changes in the structure of the HDPE after immersion in B100, with only a modest increase in the degradation temperature of the polymer occurs after exposure to the fuel.

**Figure 7:**
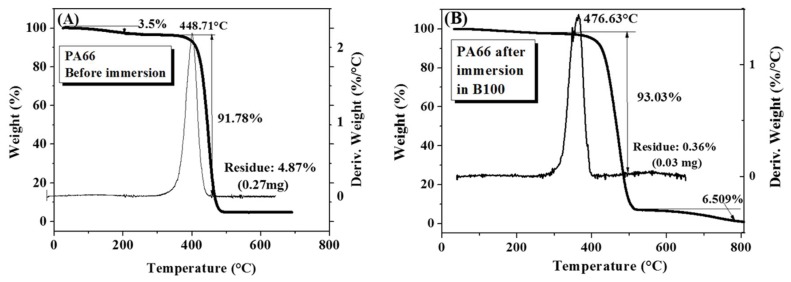
TGA curves of the PA66 samples. (**A**) Before immersion; (**B**) after immersion in B100.

Figure 7A,B present the TGA thermograms for samples of PA66 before and after immersion in B100, respectively. A small mass loss of 3.5% is initially observed around 260 °C, which is associated with the loss of the moisture that these materials usually contain [35]. This relative high temperature corresponds the temperature in which physisorbed water molecules evaporate. It is known that the hydrogen bonds can be formed between water and the amide groups from the polymer chain. Before the immersion, a decomposition of PA66 takes place at 448.71 °C, with a mass loss of 91.78% and a final residue of 4.86% that is attributed to the presence of fillers. After the immersion, a decomposition of the polymer occurs at 476.63 °C, which is a greater temperature than the one observed in the sample before immersion. The mass loss and the residue were 93.03% and 0.36%, respectively. These results indicate that the thermal stability of PA66 slightly increases after exposure to biodiesel. By comparing thermograms of the samples before and after immersion in B100, it can be stated that the fuel did not affect the thermal stability of the PA66, since there were no significant changes in the degradation temperatures or in the initial mass loss of the polymer.

**Figure 8:**
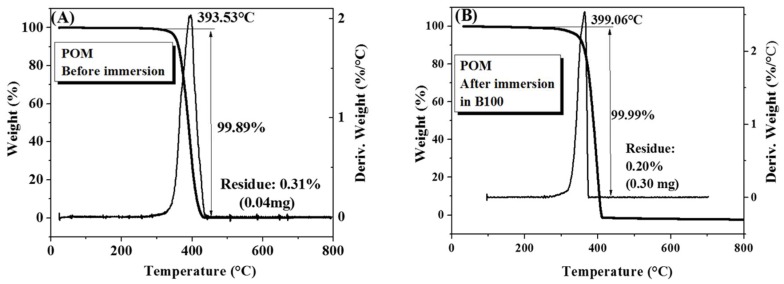
TGA curves of polyoxymethylene (POM) samples. (**A**) Before immersion; (**B**) after immersion in B100.

**Figure 9:**
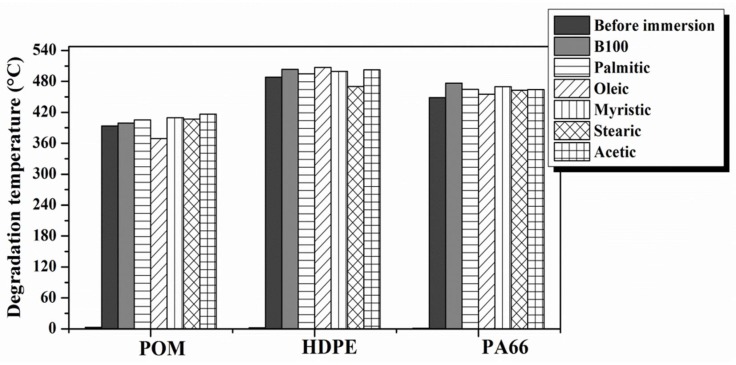
Degradation temperature extracted from thermogravimetric analyses performed on polymer samples before and after exposure to B100 and B100–acid blends.

Figure 8 shows thermogravimetric curves and their derivatives for POM samples before and after immersion in B100. A significant mass loss of 99.89% was observed at a maximum temperature of 393.53 °C (Figure 8A) [36]. At the end of the test, a residue of 0.31% was obtained. On the other hand, after exposure to the B100, these samples exhibited higher thermal stability than the pristine, having a mean degradation temperature of 399.06 °C with a mass loss of 99.99% and a residue of 0.29% (Figure 8B). From the thermogravimetric results, it can be stated that there were no significant changes in the thermal stability of POM after exposure to the fuel, except for the small changes discussed above. TGA analyses were also carried out on polymer samples before and after exposure to B100–acids blends. The aim of this trial was to evaluate if the addition of acid to the B100 has an influence on the thermal degradation of the polymers. A compilation of these results is shown in Figure 9.

The POM samples exhibited a slight increase in degradation temperature when exposed to the B100 and B100–acid blends, except in oleic blends in which a decrease of degradation temperature was observed. This could be related to the larger unsaturation degree of the oleic acid that makes oleic acids an easily oxidizable compound [37,38]. The oleic acid can react with oxidizer species present in the biodiesel, generating products such as aldehydes, ketones, alcohols, water, and esters. These products can be absorbed by the POM and induce a decrease of degradation temperature. The absorption of oxidized products into the POM can be explained by the fact that this polymer has partial polarity [33], so, it can absorb polar compounds present in the oxidized biodiesel during the exposure period (see Figure 2).

The HDPE samples exhibited an increase of the degradation temperature in all fuels, except when exposed to B100–stearic acid blend. HDPE showed a greater increase in weight in the mixture of biodiesel with stearic acid (see Figure 2), which may be associated with the decrease of the temperature of degradation of this material. Corrosion of plastics can be classified in the following way (according to type of attack): absorption, permeation, and solvent action [33]. In the current case, the absorption of B100–stearic acid blend by HDPE causes a decrease in its degradation temperatures. When the polymer (HDPE) and the permeant (B100–stearic acid blend) both have similar functional groups, the permeation rate will increase [33]. The stearic acid is one of the fatty acids with the highest number of carbons in the nonpolar aliphatic chain that makes it affine with the HDPE. The lower the intermolecular chain forces (van der Waals) of polyethylene, the higher the permeation rate [33]. The PA66 samples exhibited a slight rise in degradation temperature in all test media. The slight increase in its degradation temperature and the rise in its thermal stability observed after exposure to all the fuels can be explained by the formation of strong secondary links that interact with the polar groups present in the biodiesel [33]. However, the addition of acid compounds to B100 does not induce significant changes in the degradation temperature of the polymer.

### 3.4. FTIR Analysis

**Figure 10:**
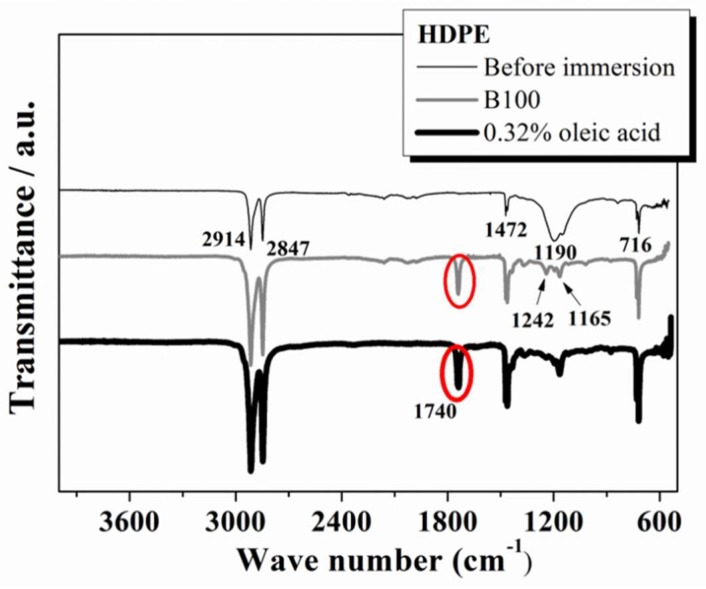
Fourier transform infrared (FTIR) spectra for the HDPE samples performed before and after immersion in B100 and B100–oleic acid blend.

Figure 10 shows the FTIR spectra for the HDPE evaluated before and after immersion in B100 and B100–oleic acid blend. The FTIR spectrum of the HDPE before immersion shows strong absorption bands at 2914 and 2847 cm^−1^, corresponding to the symmetric stretching of the C–H group [39]. The bands at 1472 and 716 cm^−1^ correspond to the deformation of the tertiary carbon [39]. The broad band at 1190 cm^−1^ corresponds to the fluorine content in the HDPE evaluated [40].

**Figure 11:**
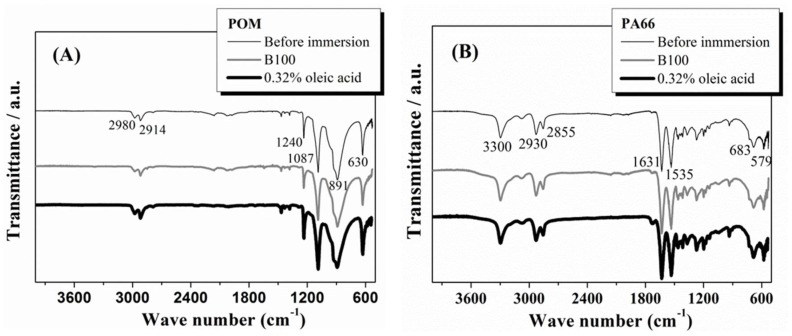
FTIR spectra for the (**A**) POM and (**B**) PA66 samples performed before and after immersion in B100 and B100–oleic acid blend.

The HDPE samples after immersion show a new band at around 1740 cm^−1^, corresponding to the stretching of C=O, which is assigned to an aldehyde group and an ester group which are mutually superimposed [21]. It can be deduced that this band comes from the irreversibly adsorbed biodiesel by the polymer. Additionally, the broad band at 1190 cm^−1^ is substantially reduced in intensity and transformed into two well-defined bands at 1242 and 1165 cm^−1^ when the polymer was exposed to B100 and B100–acid blends. This occurs as a consequence of the interaction with the methyl esters of fluorine groups present in the biodiesel. Therefore, the bands that appear between 1190 and 1300 cm^−1^ were assigned to the C–F stretching trifluoromethyl (CF_3_) group [41,42]. The stretching band of the CF_3_ group was assigned to the peak observed at 1242 cm^−1^. Additionally, the asymmetric stretching mode of the fluoromethyl group was observed at 1165 cm^−1^ [39]. Similar spectra of the HDPE after exposure to the other acid blends were recorded (not shown in this paper) and the results were analogous to those observed for exposed HDPE to B100 and B100–oleic acid blend. The fact that no additional changes in the FTIR spectra were given by the acid addition to B100 suggests that the addition of acids to B100 does not induce additional chemical or structural changes to the HDPE after immersion into the blends. FTIR spectroscopy was also performed on samples of POM and PA66 polymers before and after immersion in B100 and B100–acid blends. The FTIR spectra of POM are shown in Figure 11A. The typical strong absorption bands for this material can be observed at 891 and 1087 cm^−1^ due to stretching of the C–O–C bonds, and moderate bands at 630, 1240, 2914 cm^−1^ due to stretching of the CH_2_ [43,44]. No significant changes of the spectra were observed in the POM samples after immersion. Likewise, the addition of acid to B100 did not induce additional variation of the FTIR spectra of the polymer. These results are consistent with the results of mass variation and thermogravimetric analyses of the POM, where no important changes were observed after immersion, indicating weak interaction between POM and the fuels. Finally, the FTIR spectra of the PA66 (Figure 11B) before and after immersion show the following characteristics bands for this material: at 3300 cm^−1^ (the symmetric stretching of NH), at 2930 cm^−1^ (CH group stretching), at 1631 cm^−1^ (amide I, stretching of C=O), and at 1535 cm^−1^ (amide II, CN group stretching) [45,46,47]. For PA66 polymer, no important changes in the chemical structure of the samples evaluated by FTIR before and after exposure to the fuels were observed. This indicates that the chemical structure of the PA66 polymer is not affected by exposure to the biodiesel and acid blends. The observed changes in the degree of crystallinity of the PA66, evidenced by the DSC thermograms, are not due to changes in the chemical structure of the material, but rather to a rearrangement of the molecular chains of the polymer, evidenced also by changes in its crystallinity.

### 3.5. Evaluation of the Mechanical Properties

**Figure 12:**
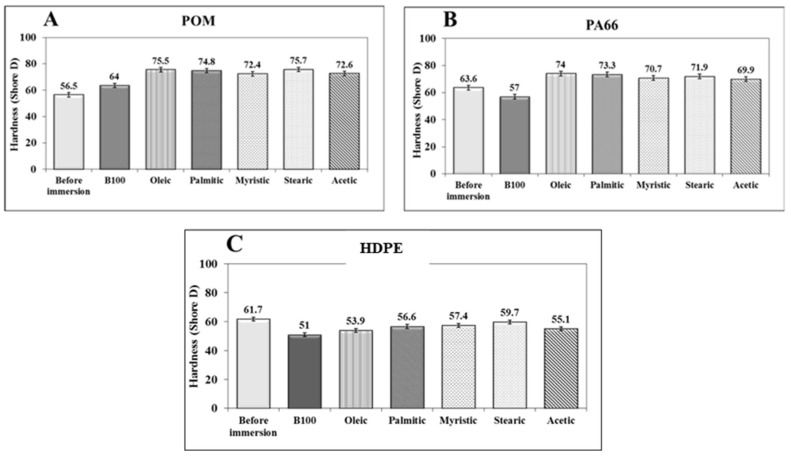
Hardness variation of the polymeric materials after immersion in B100 and B100–acid blends. (**A**) HDPE, (**B**) PA66 and (**C**) POM.

Figure 12 shows the variation of the hardness values of the polymeric materials tested before and after immersion in B100 and B100–fatty acid blends. The hardness of the POM samples immersed in B100 and B100–fatty acid blends increased by about 13% and 33%, respectively, in comparison to those specimens not exposed to the fuels (see Figure 12A). The hardness increase of the polymer could be related to the rise in its crystallinity according to Askeland [48]. Although the increase in the degree of crystallinity of the POM after immersion in the fuels was not significant (around 7% in B100–fatty acid blends), it could be enough to raise the hardness of the polymer. The increase in crystallinity promotes a more cross-linked structure, which leads to rigidity and to an increase in the hardness. Similar behavior was observed in the variation of hardness of PA66 samples after immersion in B100–fatty acid blends. As can be seen in Figure 12B, an increase in the hardness of the PA66 was observed after immersion in the fuels. This is related to the increase of the degree of crystallinity of the polymer after immersion in the fuel blends, as was observed in Figure 5. The polymer increases its rigidity and hardness as consequence of the increase in crystallinity. On the other hand, the immersion of PA66 samples in B100 promoted a slight fall in its hardness (from 63.6 to 56.9 Shore D), whereas its crystallinity was increased (see Figure 5). This behavior can be associated with a loss of tenacity promoted by the leaching of plasticizers, as was evidenced by the small reductions in the apparent weight shown in Figure 2. In contrast to what was observed for POM and PA66 polymers, the HDPE samples exhibited a reduction in hardness values after immersion in B100 and B100–fatty acid blends, (Figure 12C). This behavior is also explained by the diminution in crystallinity suffered by the HDPE samples after exposure to the fuels (B100 and B100–fatty acid blends), as was observed in DSC analysis.

**Figure 13:**
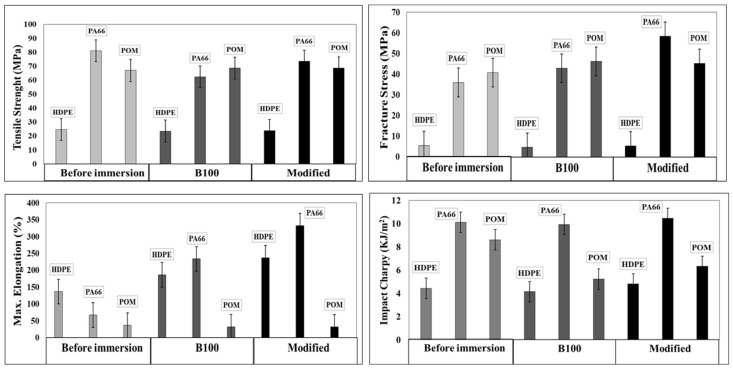
Evaluation of tensile strength, fracture stress, maximum elongation, and Charpy impact of HDPE, PA66, and POM before and after immersion in B100 and B100–fatty acid blend (modified: 0.32% palmitic + 0.32% oleic acids).

The effect of the biodiesel and biodiesel–fatty acid blends on the mechanical properties of the three polymers was determined by evaluating the changes in the tensile strength, fracture stress, maximum elongation, and impact resistance of polymers before and after immersion in B100 and B100-modified blend. As was mentioned in the experimental part, the impact resistance and stress–strain curves were performed only in B100 and B100-blend modified with the addition of 0.32% palmitic and 0.32% oleic acids (modified). Figure 13 shows the changes of the tensile strength, fracture stress, maximum elongation, and impact resistance of HDPE, PA66, and POM. The HDPE samples immersed in B100 showed a slight reduction in tensile strength and in fracture stress compared to HDPE before immersion. Additionally, the elongation of HDPE increased from 136.48% to 186.74% in B100. These results are coherent with the previous hardness results and confirm that the immersion in both B100 and B100–fatty acid blends softens the HDPE and turns it into a ductile material. The impact resistance of this material did not present significant changes after immersion in the two blends.

PA66 acquired some stiffness after being exposed to B100 and B100–fatty acid blends. This change was corroborated by the greater slope in the linear region of the stress–strain curve (elastic region, not shown in this paper) and by the increasing fracture stress values, as can be seen in Figure 13. This behavior may be related to the increase in crystallinity that the PA66 presented after immersion in the fuels. However, in the region of plastic deformation, a significant decrease of the mechanical properties was observed. Upon exposure to the blends, the PA66 presented a reduction in tensile strength, whereas it exhibited a rise in the maximum elongation in comparison with the values obtained for unexposed samples. Finally, the impact resistance of this material did not present significant changes after immersion in the fuels. The POM samples, unlike the PA66 and HDPE ones, exhibited a slight rise of tensile strength after exposure to B100 and B100-modified blend. This result is coherent with the rise in the hardness of the material observed after exposure to the fuels. The fracture stress of the POM presented a rise, while the maximum elongation and the impact resistance were significantly reduced after being exposed to the fuels. This change is associated with an increase in the stiffness of the material. These results show important changes in the mechanical properties of the material due to the interaction with the B100 and B100–fatty acid blends. The change in the stiffness of the POM samples can be associated with the change of the crystallinity of the material observed during the DSC analysis.

## 4. Conclusions

The high-density polyethylene (HDPE) samples immersed in B100 showed an increase in mass and a reduction of crystallinity associated with the absorption of nonpolar compounds present in the biodiesel and B100–acid blends. The chemical interaction between the HDPE and biodiesel seems to be related to the formation of C=O new bonds, which induce an increase in the melting temperatures of the polymer. The HDPE samples exhibited a decrement in the hardness, tensile strength, and fracture stress after immersion in B100 and B100–acid blends. Additionally, the maximum elongation of HDPE increases after immersion, becoming a more ductile material. However, the addition of acid compounds to B100 does not induce further variations of the mechanical properties of the HDPE with respect to those observed in B100.

Polyoxymethylene (POM) did not show any significant mass variations or change in thermal properties after immersion in B100 and B100–acid blends. In addition, according to FTIR analysis, no chemical interaction between POM and the B100 blends were evidenced after immersion. The variation of the mechanical properties of the POM after exposure to B100 and B100–acid blends was associated with increased stiffness of the material as result of the small fuel absorption. The variation on mechanical properties was potentiated by the addition of acid compounds to B100.

The polyamide 66 (PA66) samples exhibited slight weight loss in all B110 and B100–acid blends. Additionally, PA66 experienced an increase in its crystallinity and a rise in the temperature of degradation after exposure to B100 and B100–acid blends. The addition of acid to the B100 does not induce further variations of the thermal properties of the polymer. PA66 acquired some stiffness after being exposed in B100 and B100–acid blends, which is related to the change in the crystallinity of the material. However, in the region of plastic behavior, a significant reduction of the mechanical properties was observed, which was potentiated by the addition of acid compounds to B100.

As a final recommendation, the HDPE and POM can be used in auto part components exposed to B100; however, if the B100 exhibits signs of oxidation or the presence of acid compounds, the use of HDPE should be avoided. The use of PA66 in auto part components exposed to B100 should be avoided in all cases.

## Figures and Tables

**Table 1:** Chemical composition of the used palm biodiesel (% mass).

Type of Methylester		% Mass
Myristic	1.03	
Palmitic	43.3	
Stearic	4.20	
Palmitoleic	0.15	
Oleic	41.8	
Linoleic	9.10	
Linolenic	0.15	
Total saturated methylesters	48.8	
Total unsaturated methylesters	51.1	

**Table 2:** Physicochemical properties of the used palm biodiesel.

Property	Method	B100
density at 15 °C (kg/m^3^)	ASTM D1298	871.6
kinematic viscosity at 40 °C (mm^2^/s)	ASTM D445	4.6
higher heating value (MJ/kg)	ASTM D240	39.86
lower heating value (MJ/kg)		37.14
lower heating value (MJ/m^3^)		32,444
Rancimat oxidation stability (h)	EN 14112	12.83
cloud point (°C)	ASTM D2500	18
cetane number (mass weighted average)		63.7
average reported cetane number		62.7

**Table 3:** Physicochemical properties of the used acid compounds in the biodiesel blends.

Acid	Molar Mass (g·mol^−1^)	Density (g/mL)	Solubility in Water (mg/L)	Melting Point (°C)	Boiling Point (°C)	Flash Point (°C)
Myristic	228.38	0.99 (24 °C)	20 at (20 °C)	54.4	326.2	110
Palmitic	256.43	0.852 at (25 °C)	7.19 at (20 °C)	62.9	351	206
Stearic	284.48	0.9408 at (20 °C)	0.34 at (25 °C)	69.3	361	113
Oleic	282.47	0.89	Insoluble	14	360	189
Acetic	60.05	1.049	Miscible	16	118	40
